# Attention Deficit Hyperactivity Disorder and Substance Use Disorder: A Narrative Review

**DOI:** 10.7759/cureus.24068

**Published:** 2022-04-12

**Authors:** Bahadar S Srichawla, Chloe C Telles, Melanie Schweitzer, Bilal Darwish

**Affiliations:** 1 Neurology, University of Massachusetts Medical School, Worcester, USA; 2 Medicine, William Carey University College of Osteopathic Medicine, Hattiesburg, USA; 3 Medicine, New York Institute of Technology College of Osteopathic Medicine, Old Westbury, USA; 4 Medicine, Idaho College of Osteopathic Medicine, Meridian, USA

**Keywords:** neuropsychiatry, stimulant abuse, medicine, drug addiction, neurology, addiction psychiatry, psychiatry, opioids use disorder, substance use disorder, attention deficit hyperactivity disorder (adhd)

## Abstract

Attention deficit hyperactivity disorder (ADHD) has a growing incidence and prevalence in the United States and throughout the world, much of which is contributed to increased awareness of the condition and solidified diagnostic criteria. Substance use disorder (SUD) similarly has seen a sharp increase, particularly with the rising cases of opioid abuse. Management of ADHD is done primarily with pharmacologic therapy, often stimulants and with psychosocial interventions (i.e., exercise, meditation, peer-to-peer intervention, etc.) for adjunctive management. Management of SUD involves cessation and treatment based on the underlying drug of abuse. Many clinicians are uncomfortable treating ADHD in patients with SUD based on concerns the intervention may lead to an adverse event, including drug relapse, and the development of other psychiatric comorbidities. Concerns also arise about stimulants acting as a gateway drug in adolescents leading to the onset of SUD.

Thus, in this narrative review, we aim to shed light on ADHD in relation to SUD and to provide clinical insight based on the current scientific literature on the topic. ADHD causes lesions in subcortical structures in the basal ganglia and limbic system. Treatment of ADHD with stimulants has been shown to normalize malformed neuroanatomical variations and lead to improved long-term outcomes compared to non-treatment of ADHD. Based on current scientific literature, it is recommended to treat ADHD with guideline-directed pharmacologic agents including stimulants along with non-pharmacologic interventions primarily exercise. There may be some improvement in reducing risky behavior, such as substance abuse, and may even help prevent the development of SUD.

## Introduction and background

Epidemiology

Attention deficit hyperactivity disorder (ADHD) is diagnosed in patients who show patterns of persistent impulsivity, hyperactivity, and inattention. The Diagnostic and Statistical Manual of Mental Disorders (DSM-V) is the leading resource for physicians to classify symptoms and in creating the diagnostic criteria for ADHD. Some of these symptoms include careless mistakes in schoolwork, inability to hold a conversation, distractibility, and losing items for daily tasks (i.e., writing utensils, paper). ADHD remains a diagnosis in adolescents less than 17 years of age however, the diagnosis in adults is made based on refined criteria [1].

The prevalence of ADHD has increased substantially. Globally, 2%-7% of people have been diagnosed [2]. Within the United States specifically, there has been an increase in patients with ADHD from 6.1% in 1997-1988 to 10.2% in 2015-2016 [3]. Data from 2019 showed an incidence of ADHD in boys aged 3-17 at 11.9% compared to 5.7% in girls [4]. Studies looking at ADHD diagnosis based on patient gender show the ratio of diagnosis in school-aged children to be 7.5:1 compared to 3:1 in males versus females respectively. Conversely, the gender ratio of males versus females in adolescents was 8.1:1 to 1.6:1 at the same time [5]. Overall, it is theorized that the increased prevalence may be because of increased awareness of ADHD and its varying presentation. Parents, educators, and doctors are more aware of the condition and thus more likely to diagnose children with it. Studies looking at disease incidence based on race show a higher incidence of ADHD in Caucasian individuals and a lower incidence in African Americans [6]. This discrepancy may be because of differences in access to care, cultural acceptance, and recognition and classification of symptoms. Patients presenting with ADHD commonly have other comorbid conditions including autism spectrum disorder among other heritable psychiatric conditions. ADHD has been deemed heritable as well and is more often diagnosed in patients who have family members that share the diagnosis [6].

Adolescent substance abuse is considered to be the number one public health threat in the United States by multiple public health organizations. Adolescents diagnosed with substance use disorder (SUD) often have comorbid psychiatric conditions including depression, anxiety, and ADHD. Management of SUD involves removal of the offending agent and avoidance of medications known to increase addictive behaviors, which are often used in the management of ADHD [7]. Co-management of both ADHD and SUD continues to be an area of trepidation amongst primary care clinicians. Thus, we aim to review the literature on the relationship between ADHD and SUD and draw recommendations based on it.

Pathophysiology of ADHD

A proposed pathophysiologic mechanism for ADHD is secondary to catecholamine imbalance of dopamine (DA) and norepinephrine (NE). The prefrontal cortex (PFC) is integral for executive functions such as working memory and attention regulation [8]. The PFC is densely populated with DA and NE receptors and it communicates with the basal ganglia via the frontostriatal pathway. Impaired neurotransmission of catecholamines results in poor inhibitory control [9]. This proposed mechanism is strengthened by imaging studies conducted on patients with ADHD. Magnetic resonance imaging (MRI) in patients with ADHD has shown reduced volumes in the PFC, cerebellum, and subcortical structures [10]. Diffusion-tensor imaging has shown alterations in cortical morphology of adolescents with ADHD [11]. Functional magnetic resonance imaging (fMRI) revealed reduced local activation of the frontostriatal pathway during tasks that require attention and inhibition [10]. ADHD is characterized by diminished executive function such as response inhibition, attention, and working memory. These functions are modulated by catecholamine transmission between the PFC and basal ganglia. Abnormalities in imaging as described help substantiate the proposed mechanism for ADHD.

ADHD management

Pharmacologic management for ADHD is categorized into two categories: stimulants and non-stimulants. The mechanism of action for stimulants includes a synaptic blockade of the reuptake of catecholamines such as dopamine (DA) and norepinephrine (NE) and the direct release of catecholamines into the synaptic cleft leading to increased neurotransmission. Examples of such stimulants include amphetamine/dextroamphetamine and methylphenidate. Non-stimulants function as NE reuptake inhibitors or as alpha 2 agonists. Atomoxetine, viloxazine, guanfacine, and clonidine are categorized as non-stimulants. Atomoxetine and viloxazine are NE reuptake inhibitors. Guanfacine and clonidine are alpha 2 agonists [12]. 

Non-pharmacologic treatment modalities are available for ADHD as alternatives to pharmacotherapy but there is limited evidence to suggest substantial efficacy. Examples of holistic modalities include, but are not limited to, physical exercise, changes in diet, various supplements (i.e., fatty acids, vitamins, St. John’s wort, other herbs, and minerals), mindfulness, and biofeedback techniques. Unlike FDA-approved pharmaceutical products, most alternative treatments for ADHD have not been studied in randomized controlled trials. Digital health interventions such as wearables and smartphone apps may also hold potential as non-pharmacologic interventions [13]. Evidence-based guidelines do not suggest routine intervention with non-pharmacologic treatments for children with ADHD [14].

SUD management

Management of SUD in adolescents requires close communication between the individual, physician, and family. Psychosocial interventions continue to be the main-stay management of SUD. These interventions are mediated through family-oriented therapy and cognitive behavioral therapy. Many of these interventions hope to uncover underlying triggers and social pressures that promote young individuals towards substance abuse. And by addressing these concerns, cessation of usage can be achieved [15]. 

Supportive management is indicated and has shown some efficacy in being useful. Other interventions include mindfulness-based meditation and exercise; however, further research is needed in this field to assess long-term abstinence. Pharmacologic-based intervention for SUD is substance-specific. For example, managing nicotine abuse is best done with nicotine replacement therapy. Alcohol abuse with naltrexone or acamprosate, and opioid abuse through buprenorphine and methadone. Selective serotonin reuptake inhibitors (SSRIs) are also used in SUD with comorbid psychiatric conditions [16]. Cases of stimulant abuse including cocaine, methamphetamine abuse, etc. are best managed with cessation of the underlying drug and GABAergic medications (i.e., benzodiazepines) [16].

With the rising cases of ADHD and the increasing prevalence of SUD, co-managing these two conditions continues to be an area of difficulty amongst some clinicians. We aim to give insight into evidence-based practices that have shown adequate co-management of both conditions and to shed light on the relationship between ADHD management and substance abuse disorder.

## Review

We completed a literature search using PubMed/MedLine/PubMed Central with broad search terms related to ADHD and SUD. Further search terms included epidemiology, screening, pathophysiology, pharmacologic and non-pharmacologic interventions. No specific inclusion and exclusion criteria were utilized. Articles included original research, meta-analyses, and mixed systematic and meta-analyses. The review was completed with adherence to the Scale for the Assessment of Narrative Review Articles (SANRA) guidelines.

Comorbidity of ADHD and SUD

The development of neuropsychiatric conditions like ADHD has long been considered a diagnosis of adolescents and may be correlated with childhood adversity. Similarly, SUD presenting in the adolescent years has had some correlation with adverse events in childhood. However, more research is needed on this hypothesis [17,18]. One meta-analysis in 2012 showed amongst individuals diagnosed with ADHD 23.1% had a diagnosis of SUD (21% in adults and 25.3% in adolescents) [19]. Almost one in four individuals shared the diagnosis of ADHD and SUD. The comorbid diagnosis of both conditions warrants further analysis of the complex psychosocial aspects and the effects of management on one another.

Screening for ADHD

Screening for ADHD in both childhood and adulthood is done using clinical algorithms and is one weapon in a clinician’s arsenal to tackle ADHD. One such algorithm that can be used in the screening for ADHD is the World Health Organization’s Adult ADHD Self-Report Scale (ASRS). The ASRS bases its criteria for screening on the Diagnostic and Statistical Manual (DSM-V) and is the most common algorithm used in the diagnosis of ADHD. A recent study pooling patients from both the United Kingdom and the United States showed overdiagnosis of ADHD based on the ASRS criteria. The study revealed an approximate 7-10 times over-prediction in the diagnosis of ADHD. Thus, the authors of this study point toward using accurate clinical assessments and controlling for other psychiatric conditions which may show increased impulsivity [20]. 

Patients who screened positive for ADHD using the ASRS criteria were also found to have an increased prevalence of comorbid psychogenic non-epileptic seizures (PNES) compared to epileptic seizures in the epilepsy monitoring unit (EMU) [21]. This provides further compounding evidence that ADHD is often comorbid with other psychiatric conditions. ADHD is the most common behavioral condition diagnosed in children. Physicians should recognize the need for screening for ADHD based on behavioral complaints and poor academic performance. Recognizing chronicity and triggers for these patterns of behavior and symptoms also is important. Many acute stressors in a child’s life can cause mood disturbances, lack of motivation, inattention, and irritability in children which may prematurely lead to a diagnosis of ADHD. Whenever these acute stressors are identified it is important for the clinician to recognize and remove this stressor before initiating pharmacotherapy for ADHD. In the setting of true ADHD, both psychosocial intervention and pharmacologic therapy are recommended [22]. 

(SUD) is a major public health threat in adolescents throughout the world. Screening for substance abuse in adolescents is imperative for the physician in improving morbidity and mortality in young people. Multiple screening tools have been used for this purpose. Commonly used screening tools include CRAFFT and DAST questionnaires. The utilization of these questionnaires aims to recognize substance abuse more readily with the hope of earlier intervention. The Screening, Brief Intervention, and Referral to Treatment (SBIRT) strategy was developed in the United States to tackle the immense public health threat that SUD poses [23]. Recent studies have shown high rates in the utilization of SBIRT with some variation from center to center. Larger trials assessing the overall benefit in morbidity and mortality with SBIRT utilization are needed [24].

Specific pharmacologic treatment strategies for ADHD

Treatment recommendations for ADHD in children differ based on age group (i.e., preschool-aged children, school-aged children, and adolescents). Current guidelines do not recommend diagnosing ADHD below the age of four. Symptoms typically develop during school-age (ages 6-12). Diagnostic workup initially should include evaluation for both psychiatric stressors and medical conditions. These acute stressors commonly affecting children at this age include tense interpersonal relationships between siblings and marriage difficulties between parents. The diagnosis of ADHD in adolescents greater than the age of 12 is less common, therefore physicians should be vigilant when screening for these acute stressors including school bullying and depression [21]. First-line treatment for ADHD in preschool-aged children (4-5 years old) is behavioral therapy administered by parents or teachers. Second-line treatment is methylphenidate and targets situational factors (e.g., expulsion from school, risk of harm to others, strong family history of ADHD, central nervous system injury). Guidelines suggest that first-line treatment for school-age children (ages ≥6) is stimulant medication combined with behavioral therapy. Non-stimulant medications may be used for some children [25]. Overview of the diagnostic and management guidelines of ADHD in children is stratified based on age as identified in Table 1.

**Table 1:** ADHD management and workup strategy stratified based on age.

Age group	Diagnostic and management strategy
Preschool Children (Age 4-5)	Behavioral therapy administered by parents or teachers.
Children (Age 6-12)	Medical management with consideration for comorbid learning disorders, and sleep disorders (i.e. obstructive sleep apnea, adenotonsillar hypertrophy, ADHD).
Adolescents (Age >12)	Medical management primarily with stimulants, peer-to-peer interventions, behavioral therapy. Recognizing acute stressors, sleep disorders, etc.

As clinicians, it is essential to consider the efficacy of various medications available for ADHD, including methylphenidate, amphetamines, and non-stimulants. Methylphenidate is most efficacious with reduced hyperactivity, impulsivity, and inattention in dosages around 0.3 to 0.6 mg/kg. It is available as a tablet, chewable tablet, or liquid. After six months, a discontinuation trial is recommended to reassess the severity of symptoms. Amphetamines are classified as single salt dextroamphetamine or amphetamine sulfate or as a mixed dextroamphetamine-amphetamine salt. It is available in tablets and oral solutions. Another amphetamine is lisdexamfetamine, a prodrug of dextroamphetamine. Formularies for lisdexamfetamine include immediate and sustained release (i.e., capsules, liquid suspension, tablets, oral disintegrating tablets, chewable tablets) [26].

Although highly efficacious for many patients, methylphenidate and amphetamines bear notable side effects. Common side effects are poor growth during childhood, weight loss in childhood, loss of appetite, sleep deficits, psychomotor irritability, and emotional disturbances. Mixed dextroamphetamine-amphetamine salts are associated with less weight loss but an increased likelihood of irritability when compared with methylphenidate. Less common side effects include, but are not limited to, increased heart rate, increased blood pressure, headache, dizziness, gastrointestinal discomfort, priapism, and peripheral vasculopathy. Preschool-aged children are at an increased risk of experiencing side effects than school-aged children. Contraindications to stimulant use in children and adolescents with ADHD may be considered when analyzing the risk of such stimulants. Contraindications include, but are not limited to, history of drug abuse, symptomatic cardiovascular disease, hypertension, hyperthyroidism, anxiety, motor tics or Tourette syndrome, glaucoma, and concurrent use or use within two weeks of monoamine oxidase inhibitors [26,27].

Alpha-2-adrenergic agonists, clonidine and guanfacine, are commonly used non-stimulant treatment options for ADHD. These medications are therapeutic for children with ADHD who need an adjunct to stimulant therapy or after stimulants and/or atomoxetine have failed. Clonidine can be prescribed in an extended-release form for children and adolescents aged six to 17 years [28]. Adjunctive clonidine for ADHD may also function to reduce comorbid tics or Tourette syndrome. One possible disadvantage is that the initial therapeutic response may take about two weeks compared to short-acting stimulant therapy. Advantages of alpha-2-adrenergic agonists include no known abuse potential, and that clonidine is not a controlled substance. In addition, clonidine’s potential side effects of hypotension, sedation, bradycardia, and depression may help reduce some of the side effects associated with stimulant therapy.

Guanfacine may also be prescribed for adolescents aged six to 17 years as primary or adjunctive therapy to stimulants for ADHD. Guanfacine has fewer side effects and a longer half-life than clonidine. Both extended release and immediate release are available. However, guanfacine is still likely to produce a rebound increase in blood pressure once discontinued and should be tapered. Four randomized, double-blind studies have shown that extended-release guanfacine is efficacious and well-tolerated compared to placebo when treating ADHD in children and adolescents, including those with the comorbid oppositional defiant disorder (ODD) [29-32]. Combined methylphenidate and guanfacine may improve working memory compared to guanfacine alone, but methylphenidate alone still revealed the most improvement in ADHD symptoms [33]. Guanfacine may be trialed for patients with ADHD and comorbid tics who experience worsening tics with stimulants and/or excessive sedation with clonidine [28]. Pharmacologic agents indicated for ADHD are shown in Table 2.

**Table 2:** Pharmacologic interventions indicated for ADHD management with mechanism and adverse effects.

Drug	Mechanism of action	Side effects
Methylphenidate	Norepinephrine and dopamine reuptake inhibitor (NDRI)	Poor growth during childhood, weight loss in childhood, loss of appetite, sleep deficits, psychomotor irritability, and emotional disturbances
Amphetamines	Increases neurotransmitter synthesis, vesicular monoamine transporter 2 (VMAT-2) inhibitor	Poor growth during childhood, dysgeusia, loss of appetite, sleep deficits, nervousness, and emotional disturbances
Clonidine	Alpha-2-agonist	Anxiety, chest pain, palpitations
Guanfacine	Alpha-2-agonist	Weakness, headache, dry mouth, stomach pain.
Atomoxetine	Selective norepinephrine reuptake inhibitor (SNRI)	Heartburn, loss of appetite, constipation, weight loss.

Non-pharmacologic treatment options for ADHD and SUD

Non-pharmacologic interventions proposed for the management of ADHD include mindfulness-based stress management, exercise, and meditation amongst others. Pharmacologic management of ADHD remains the long-term management option given its proven benefits, however, using non-pharmacologic interventions can be used for adjunctive management [9,14]. One study looking at the cognitive effects of exercise and ADHD found a significant improvement in executive function regardless of the physical activity done [34]. One study looking at the effects of yoga on children with ADHD showed inconclusive evidence based on its efficacy [35]. Another study looking at body-oriented mindfulness activity showed some benefits in managing ADHD symptoms [36]. Overall, a multitude of non-pharmacologic interventions can be considered; however, larger studies are needed to prove their usefulness. A meta-analysis looking at non-pharmacologic interventions for ADHD from 1980 to 2017 showed they are best used in conjunction with pharmacologic management [37]. Non-pharmacologic interventions have long been adjunctive therapies for individuals suffering from SUD. Similar to ADHD, exercise has shown improvement in long-term outcomes related to SUD [34]. Further research is needed on adjunctive therapies including mindfulness-based practices and meditation in SUD.

Long-term outcomes of untreated ADHD

**Figure 1:**
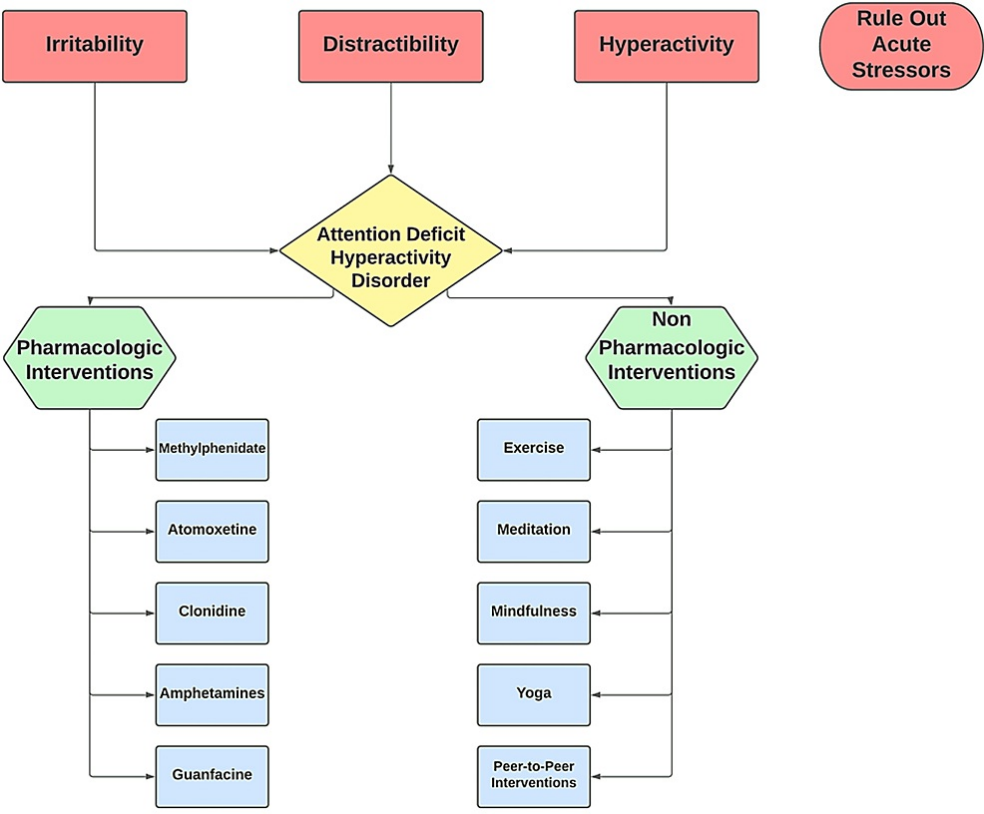
Symptoms and management strategies for ADHD.

ADHD remains an underrated and under-treated condition. ADHD, when left untreated, has been shown to cause increased risky behavior, motor vehicle accidents, frequent breakups, and substance abuse [20]. It can be hypothesized that these adverse events may result from long-term neuroanatomical effects of ADHD on the pre-frontal cortex and limbic system as previously described. Thus, the recognition of ADHD is important in childhood to prevent adverse events later in adulthood. Given the high comorbidity with other psychiatric conditions, it is recommended that treatment of ADHD begin promptly. Earlier recognition and treatment of ADHD have the potential to significantly impact morbidity in later adulthood [38]. One analysis looking at individuals with untreated ADHD compared to treated ADHD showed that non-treatment of ADHD led to the worst long-term outcomes. Notably, an increase in substance abuse was noted in the unmanaged ADHD group [39]. Figure 1 illustrates the overarching approach to ADHD.

Relationship between pharmacologic management of ADHD and SUD

There is significant discussion about whether or not treating patients (especially children) with ADHD can lead to substance abuse later in life. This concern arises because methylphenidate, the most common medical treatment for patients with ADHD, has many similarities to other addictive medications such as cocaine and methamphetamines [40]. Some literature suggests ADHD and SUD can occur co-morbidly. However, the causality of the two conditions is uncertain [41]. Individuals who have both ADHD and psychosis have a greater risk of SUD when compared to those with ADHD alone [42]. One meta-analysis reported amphetamines to be most efficacious in adults and methylphenidate in children for managing ADHD. The efficacy in children was measured by ADHD core symptoms reported by clinicians and teachers [43,44]. An additional meta-analysis in 2015 concluded methylphenidate may improve teacher-reported behavior, parent-reported quality of life, the overall symptom severity of ADHD, and reported no evidence of increased risk of serious side effects. The magnitude of the effects cannot be determined because of the short duration of previous studies [45].

In a sample of 900 college-aged participants, misuse of stimulants was found to be correlated with ADHD symptom severity. In this study, participants with ADHD were 2.90 times more likely to engage in stimulant misuse. Only 31% of individuals with ADHD possessed a stimulant prescription. Therefore, prescription status did not seem to play a significant role in increasing the likelihood of substance misuse. In addition, the results controlled for symptoms of the ODD, a common culprit linked to substance abuse [46]. Notably, ODD is often comorbid with ADHD. Therefore, it may become difficult to show whether the vulnerability to misuse substances derives from ADHD or ODD (or other psychiatric disorders). Future studies may consider controlling for ODD and other disorders when studying the correlation between ADHD medication and SUD.

Lacy et al. revealed an increased likelihood of self-administering cocaine in adolescent rats who previously received amphetamines. After observing this behavior in rats, the researchers concluded that patients on ADHD pharmacotherapy may present with an increased risk for substance misuse [47]. Thus, there is evidence to show a link between ADHD and SUD and that both influence each other. However, further translational research is warranted to show firm evidence for a causal link between ADHD and SUD.

A meta-analysis evaluating fMRI studies found that long-term stimulant medication was associated with normal basal ganglia function [10]. Thus, providing neuroanatomical evidence to support the use of stimulants in the long-term management of ADHD. A meta-analysis by Witens et al. found that long-term use of stimulants in children for ADHD was associated with decreased substance abuse later in life. A similar study found some evidence that effective ADHD treatment can help prevent cigarette smoking [48]. Beginning ADHD pharmacotherapy at a younger age has also been associated with the prevention of SUD [49]. We hypothesize that stimulant usage in ADHD patients leads to improved neurotransmission in both the basal ganglia and limbic structures including the reward center, which may help prevent substance abuse. However, further studies are needed to support this.

## Conclusions

Screening for ADHD is important however, improved screening criteria are recommended as overdiagnosis of ADHD based on ASRS guidelines has been noted. Non-managed ADHD poses significant adverse long-term outcomes including a greater prevalence of substance abuse. Non-managed ADHD has also shown long-term neuroanatomic alterations which may be irreversible. Current recommendations include using both pharmacologic and psychosocial interventions in the management of comorbid ADHD and SUD. The scientific literature does point in favor that stimulant usage in ADHD may help prevent the development of SUD. This is further supported by fMRI showing improved neuroanatomical function within subcortical structures notably the limbic system. Larger clinical trials looking at neuroanatomical structures in patients on pharmacologic therapy for ADHD with and without SUD are needed. Further studies looking at the long-term reduction in morbidity with control for other psychiatric conditions especially ODD are also needed.
